# Research on Sustainable Development of Competitive Sports in China Based on PSR and DEA Model

**DOI:** 10.3389/fpsyg.2022.925909

**Published:** 2022-07-27

**Authors:** Su Zhang, Tong Ouyang, Shou-Yu Wei, Bei-Bei Liang, Jia-Ming Zhu

**Affiliations:** ^1^Department of Physical Education, Anhui University of Finance and Economics, Bengbu, China; ^2^School of Finance, Anhui University of Finance and Economics, Bengbu, China; ^3^School of Statistics and Applied Mathematics, Anhui University of Finance and Economics, Bengbu, China

**Keywords:** competitive sports, sustainable development, comprehensive evaluation, DEA model, PSR model

## Abstract

From the 2008 Beijing Olympic Games to the 2022 Beijing Winter Olympics, the sustainable development of competitive sports has become more and more popular in China. Therefore, for the sustainable development of China's competitive sports, first, the logical relationship of “pressure-state-response” is adopted to select the index system, covering the elements of the economy, society, policy, and so on. Principal component analysis and entropy weight method are used to construct the comprehensive evaluation model of the sustainable development of competitive sports. Second, through the coupling coordination method to study the coordinated development of China's competitive sports and economic society, and then from the perspective of the obstacle factor diagnosis method to determine the obstacles to the sustainable development of China's competitive sports system. Finally, the DEA model is used to predict the development level of competitive sports in China's provinces in the next 10–20 years. The research shows that the overall development level of competitive sports in China is good, but there are certain differences among different regions. Meanwhile, from the forecast results, the development level of competitive sports in Hainan, Shanxi, Anhui, Jiangxi, Inner Mongolia, Gansu, and Ningxia may be greatly improved in the future. Based on the above research conclusions, this study puts forward some suggestions to give full play to the joint and synergistic development effect among different regions, reasonably draw lessons from the advanced experience of competitive sports development at home and abroad, and scientifically construct the comprehensive development system of competitive sports. At the same time, the research of this study provides some reference value for the sustainable development of China's competitive sports and the coordinated development of China's competitive sports and economic society.

## Introduction

With the rapid development of society, there are many standards to measure the development level of a country, among which competitive sports is one of them (Hou and Xiao, 2021). General Secretary Xi Jinping has mentioned many times in his public speeches that the relationship between sports and economic society should be treated with dialectical thinking. Sports are not only a sport but also a source of power to promote economic and social development and a key productive force for social wealth (Liu, 2011). In the process of economic development in today's society, personal demand for sports is becoming more and more abundant, and this trend has gradually derived a series of niche sports, thus driving the growth engine of the social economy. General Secretary Jin Ping stressed that “sports are an important social cause in today's society, and the competitive sports industry is an important support for building a strong sports country, and a key component of sustainable economic and social development (Qu and Gao, 2022)”. It can be seen that the development of competitive sports is closely related to the economic development of a country.

In recent years, to promote the sustainable development of China's competitive sports, China has implemented a series of relevant policies. In 2019, China issued the Outline of Building a Strong Country in Sports, emphasizing the need to enhance the overall strength and international influence of competitive sports (Wang, 2013). In 2020, the National Sports Policy and Regulations Planning Work Key points in 2020 was issued, which clearly proposed to comprehensively promote sports reform and strengthen the construction of sports rule of law. In 2021, it issued the Guiding Opinions on Accelerating the Development of the Sports Industry, stressing efforts to develop the sports competition and sports performance market and build influential and distinctive event brands (Du et al., 2020). In the same year, in the 14th Five-year Plan for Sports Development, China clearly stated that competitive sports should be brought to a new level and the legal level of sports should be further improved. In 2022, the state issued the Main Points of Work on Mass Sports in 2022, stressing the need to promote the formation of a higher-level public service system for national fitness and push the cause of national fitness to a new level with a more ambitious attitude (Zou and Tian, 2020).

**Figure 1:**
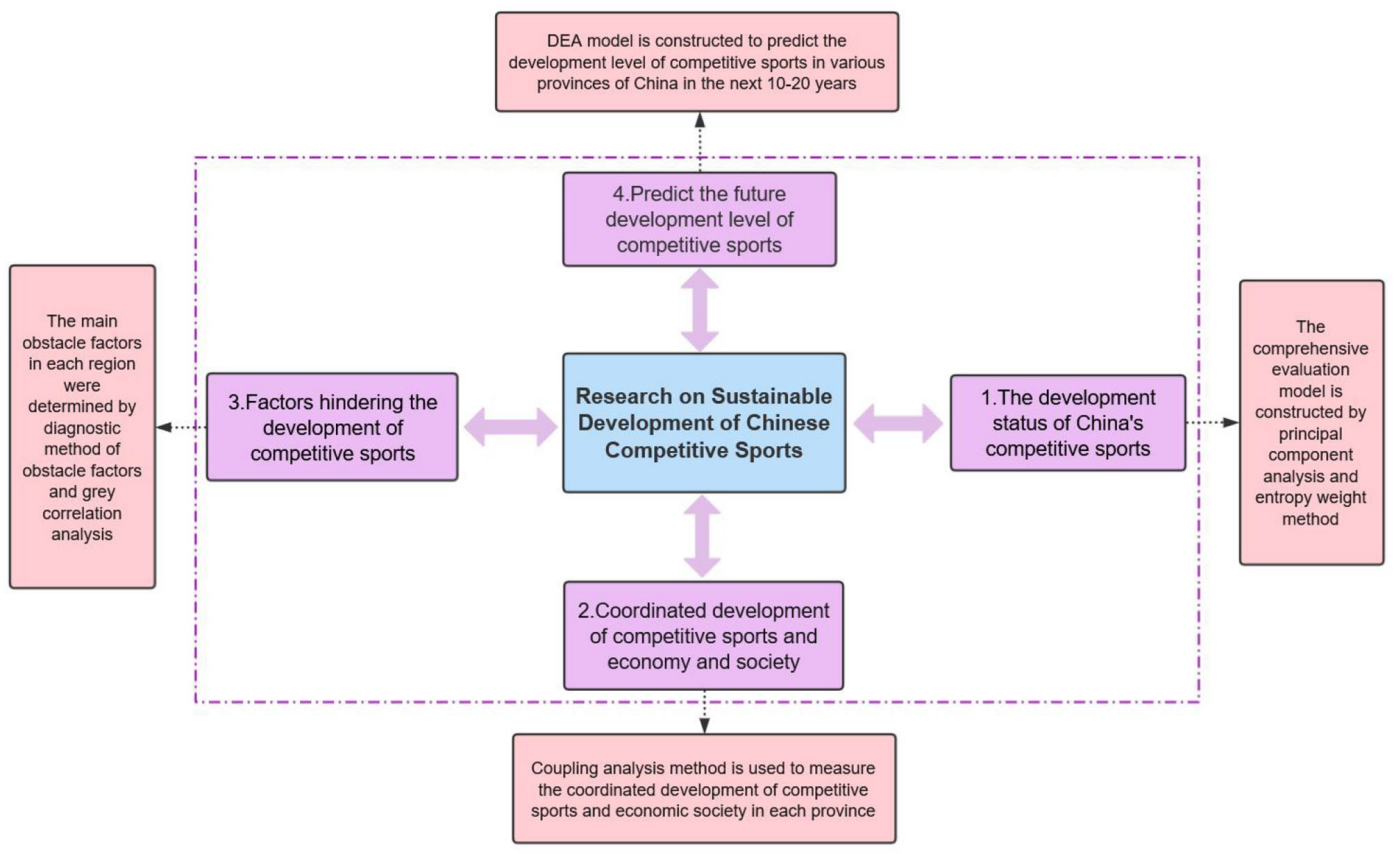
The overall research framework of the study.

With the attention of the state, China's competitive sports have been developing continuously. From the previous Olympic Games, China has made a number of achievements, China's competitive sports career has gradually made breakthroughs in various sports, and the 2022 Beijing Winter Olympic Games skiing achieving new achievements is one of them (Dong, 2015). Although the development of competitive sports is of great significance to the economic development of the society as a whole, it belongs to a huge system of competitive sports. Once the inner system is impacted by the external or internal resilience to drop, it will result in the stagnation of the whole system or collapse phenomenon, thus affecting the sustainable development of competitive sports and even the entire social undertaking (Zhou, 2019). Therefore, this study constructs a comprehensive evaluation model of sustainable development based on the current development status of the competitive sports system, studies the coordinated development of competitive sports and economic society and the hindering factors of sustainable development of China's competitive sports system, and predicts and analyzes the future development of competitive sports (Ye, 2020; Yu and Ye, 2021) to promote the sustainable development of competitive sports in China to put forward constructive suggestions. The overall research framework of this study is shown in Figure 1.

## Literature Review

### Winter Olympics and Competitive Sports

The development of competitive sports is a key part to promote the sustainable development of a country's economy and society. After the 2022 Winter Olympics, many scholars conducted research on the Winter Olympics and sports competitions (Su, 2020). Xia (2022) studied China's demeanor as a great power in the Beijing Winter Olympics under the background of sports diplomacy, demonstrating the firm belief of our country and nation in promoting the building of a community with a shared future for humanity and the good image of sunshine, prosperity, and openness, and enhancing the understanding and understanding of people around the world about China. Cui and Yin (2022) studied the effects after the Beijing Winter Olympics, believing that the Beijing Winter Olympics promoted the enlightenment value of China's ice and snow culture construction and the economic benefits of ice and snow, and contributed theoretical wisdom to the post-effect of the Beijing Winter Olympics and the development of the ice and snow industry in the post-Olympic era. At the same time, Sun et al. (2021) put forward some suggestions for selecting innovative sports talents and exploiting the comprehensive benefits of the Winter Olympics in the case of the Winter Olympics held in South Korea in the same year. In addition to playing a vital role in the Olympic Games, competitive sports are also closely related to our daily life, especially their role in rallying people and inspiring their spirits (Zhang and Sun, 2021; Zhang et al., 2022).

### Sustainable Development and Sports Competition

At present, many scholars at home and abroad have conducted relevant studies on the sustainable development of competitive sports. Yang and Peng (2018) studied the systematic structure of the sustainable development of reserve talents training in China's competitive sports and put forward the suggestion that the sustainable development of reserve talents training in China should organically unify the development degree of quantitative dimension, the coordination degree of quality dimension, and the continuity degree of mode dimension. Yang (2022) studied the difficulties in the development of competitive sports in China and put forward the countermeasures of innovative technology guidance and innovative development ideas. Peng and Yang (2021) studied the development strategy and innovation path of China's competitive sports during the “14th Five-year Plan” period and put forward suggestions to build a new model of major sports competition preparation with multiple participants of the government and social forces and focus on resolving structural contradictions of competitive sports. Wang (2019) studied the current situation of the development of competitive sports in China, deeply analyzed the strategic dilemma of competitive sports, and put forward a number of suggestions, such as gradually narrowing the regional economic gap, and formulating the combination of unity and division according to the regional characteristics. Yang and Li (2021) studied the strategic mission and innovation path of China's competitive sports in the new era and proposed that competitive sports in the new era should be “based on three new goals, shoulder four new missions, implement five new ideas and practice six new paths”. In addition, Huang (2021) used the PSR model to study the sustainable development of competitive sports in China. Han et al. studied the sustainable development of competitive sports in the “post-Olympic” period of Olympic host countries based on the theory of institutional regulation (Chun and Chun, 2011).

### DEA Model and Sports Competition

Data envelopment analysis (DEA) is a method that can evaluate multiple inputs and outputs. Its advantage lies in that it does not need to form a production function to estimate the parameters, it is not affected by the measurement units of input and output variables, and the weight in the DEA method is not affected by human subjective factors. At present, the research of the DEA model in the field of sports mainly focuses on the evaluation of the input-output efficiency of mass sports, the evaluation of the benefit of competitive sports training, and the evaluation of the financial input efficiency of sports-related industries. Based on the dynamic DEA-SBM model, Zhu (2018) studied the influence of social security input on the output efficiency of competitive sports in 30 provinces and cities from 2013 to 2016 and the differences among regions and put forward suggestions for promoting the healthy and sustainable development of sports in China. Man (2018) uses the DEA model to calculate the efficiency of coordinated operation of sports undertakings in 31 provinces of China, and constructs the system dynamics model of coordinated sustainable development of sports undertakings in China. Li and Li (2014) studied the evaluation model of competitive sports efficiency in various provinces and regions of China through the DEA model, and put forward relevant suggestions on the development of competitive sports efficiency in the low-efficiency group. You et al. (2016) used the DEA model to study the training benefits of competitive sports in various regions of China, providing a reference for decision-making departments to formulate development strategies for competitive sports. Li (2008) established a fair DEA model and measured the performance of each country in six Summer Olympics by introducing various guarantee domains of specific countries into DEA. Lei et al. (2014) treated the Summer and Winter Olympics as a parallel system and extended a parallel DEA approach to evaluate the efficiency of each participant.

Based on the above analysis, most scholars conduct qualitative research on the sustainable development of competitive sports in China as a whole, but few divide China's 31 provinces into several regions for overall and regional quantitative research and use the DEA model for prediction to draw relevant conclusions. In view of this, this study innovatively divides China into four regions according to the economic development of each province, namely east, west, central and northeast, to carry out relevant quantitative research. The possible contributions of this study are as follows: (1) according to the economic conditions of each province in China, it is divided into four regions for local and overall research to analyze the heterogeneity between regions and provide data basis for different regions to formulate relevant policies according to local conditions. (2) PSR model is used to select evaluation indicators to comprehensively evaluate the sustainable development of China's competitive sports and obtain the development status of competitive sports in different regions. (3) Study the coordinated development of competitive sports and economic society in China with the help of the coupling coordination method, and get the relationship between competitive sports and economic and social development in different regions. (4) Determine the hindering factors of sustainable development of China's competitive sports system through the diagnostic method of obstacle factors, and get the hindering degree of each factor to different regions. (5) The DEA model is constructed to predict the development level of competitive sports in each province in China in the next 10–20 years, and the future development status of competitive sports in different regions is obtained, providing empirical basis for China's relevant policies based on local conditions.

## Research on Comprehensive Evaluation of Sustainable Development of Competitive Sports Based on PSR Model

### Construction of Sustainable Development Index System for Competitive Sports

**Figure 2:**
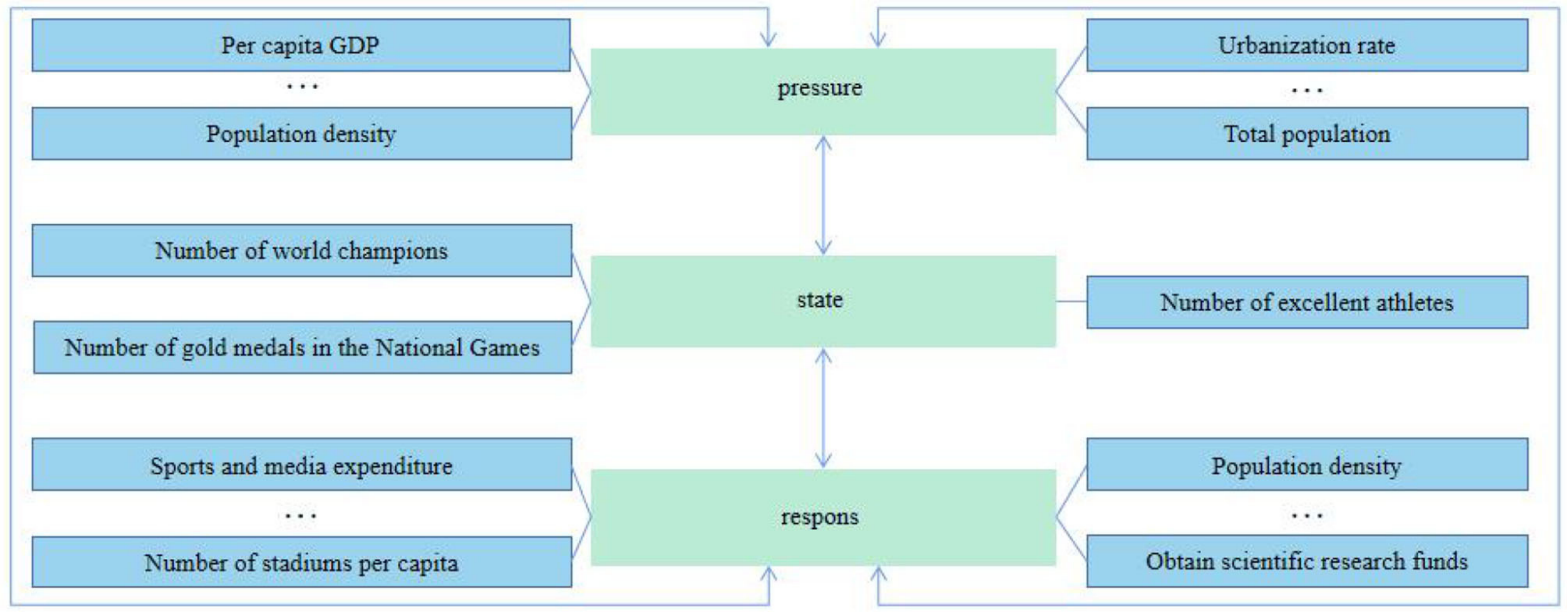
PSR model.

According to the economic development of each province, this study divides China into four regions: eastern, western, central, and northeast. In combination with the indicators selected for the sustainable development of competitive sports in China by using the PSR model in Huang's article (Huang, 2021), and the similarity between competitive sports system and natural system, the evaluation indexes of competitive sports system are determined from the logic relation of pressure-state-effect in the PSR model, and 20 second-level indexes and five first-level indexes are selected in this study. The evaluation index of the competitive sports system is determined from the logical relation of pressure—state—effect in the PSR model. At the same time, 20 secondary indicators corresponding to the three modules of “pressure-state-response” in the PSR model are selected according to the characteristics of each indicator's attributes. The PSR model is shown in Figure 2.

**Figure 3:**
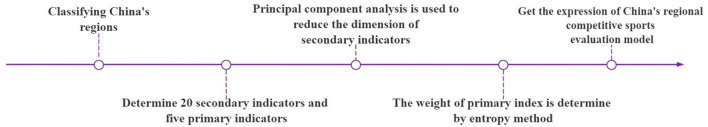
Flowchart of constructing an evaluation model.

To reasonably establish a comprehensive evaluation model for the sustainable development of competitive sports, this study performs corresponding data processing on the 20 secondary indicators that have been selected, including taking the reciprocal of the cost-based indicators, converting the data into efficiency indicators, and then standardizing the data (Cai et al., 2021; Xu et al., 2021). Then, the principal component analysis model is used to reduce the dimensionality of 20 secondary data indicators to obtain five first-level indicators. Finally, on this basis, the entropy weight method model is used to calculate the weight size, and the expression of the comprehensive evaluation model of the sustainable development of regional sports in China is obtained, and the specific process is shown in Figure 3.

This study uses 20 secondary indicators and five first-level indicators as criteria to measure the sustainable development of regional competitive sports in China. Among them, the 20 secondary indicators are per capita GDP (P1), urbanization rate (P2), total population (P3), the natural population growth rate (P4), population density (P5), the proportion of illiterate people over 15 years old (P6), the number of athletes who have won world/Olympic championships (S1), the number of gold medals in the National Games (S2), the number of athletes on outstanding sports teams (S3), the number of sports venues per capita (R1), the public budget expenditure of the sports system (R2), the expenditure of culture, sports, and media (R3), the number of full-time coaches (R4), the number of sports reserve talents (R5), the number of referee development (R6), the number of youth sports clubs (R7), the number of schools in sports traditions (R8), tertiary sector to REGIONAL GDP ratio (R9), sports lottery sales (R10), and access to scientific research grants (R11). Five first-level indicators obtained after dimensionality reduction by principal component analysis are named competitive sports pressure (CSP), competitive sports achievement (CSR), competitive sports cost (CSC), competitive sports talent (CST), and competitive sports benefit (CSB), respectively.

### Modeling and Solving

In this study, the 20 indicators of the model are tested by Kaiser-Meyer-Olkin (KMO) and Bartlett, and the test results are shown in Table 1. From the results of this test, it can be seen that the significance level of the 20 indicators of the model is high, and each indicator passes the significance test of the model, indicating that the indicators of the model can be further analyzed (Yang and Huang, 2015; He et al., 2021).

**Table 1:** KMO and Bartlett tests.

**The number of tangents for KMO sampling**		**0.601**
Bartlett spherical degree test	Approximate chi-square	606.653
	Degree of freedom	190
	Significance	0

This article calculates the values of the interpretation of the total variance for these 20 indicators and the results are shown in Table 2. Among them, the cumulative variance of components 1–5 is explained by 81.348%, which means that of the 20 components, the first five principal components can explain their total variance of 81.348%. Therefore, from the results of the total variance interpretation table, the first five principal components can be extracted as indicators after dimensionality reduction treatment (Lu and Shi, 2021; Yu and Wang, 2021; Sun et al., 2022).

**Table 2:** Explanation of total variances.

**Constituent**	**Initial eigenvalue**			**Extract the square sum of the load**		
	**Total**	**Variance percentage**	**Cumulate (%)**	**Total**	**Variance percentage**	**Cumulate (%)**
1	8.920	44.598	44.598	8.920	44.598	44.598
2	3.330	16.652	61.250	3.330	16.652	61.250
3	1.683	8.413	69.663	1.683	8.413	69.663
4	1.296	6.479	76.142	1.296	6.479	76.142
5	1.041	5.206	81.348	1.041	5.206	81.348
6	0.729	3.643	84.991	—	—	—
7	0.640	3.198	88.190	—	—	—
8	0.528	2.637	90.827	—	—	—
9	0.410	2.049	92.876	—	—	—
10	0.361	1.804	94.680	—	—	—
11	0.296	1.481	96.160	—	—	—
12	0.235	1.174	97.334	—	—	—
13	0.153	0.762	98.097	—	—	—
14	0.117	0.587	98.684	—	—	—
15	0.090	0.450	99.134	—	—	—
16	0.069	0.343	99.477	—	—	—
17	0.064	0.318	99.795	—	—	—
18	0.025	0.123	99.918	—	—	—
19	0.010	0.048	99.965	—	—	—
20	0.007	0.035	100.000	—	—	—

To be able to more reasonably determine the extracted principal component index, a factor analysis gravel plot was prepared for the 20 indicators, and the component score coefficient matrix analysis was carried out, and the analysis results were as follows.

**Figure 4:**
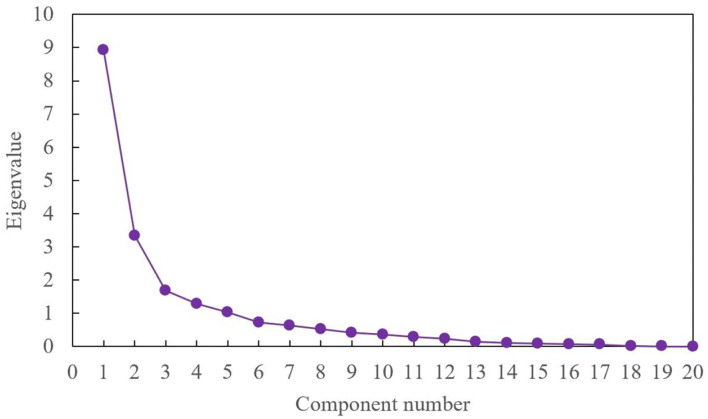
Twenty factor analysis gravel plots.

In a factor analysis lithotripsy, the vertical axis represents the eigenvalues, the horizontal axis represents the components, and the steep parts in front of the eigenvalues are large and contain more information. The polylines of factors 1, 2, 3, 4 and 5 in the Figure 4 are steep, and the subsequent folds are relatively flat and tend to be straight, and the first five factors can be extracted as common factors. Meanwhile, the score coefficient matrix values of each component are shown in Table 3.

**Table 3:** Component scoring coefficient matrix.

**Factor**	**Ingredients**				
	**1**	**2**	**3**	**4**	**5**
P1	0.21	0.3546	0.1461	−0.0537	−0.23
P2	0.1986	0.4007	−0.0218	−0.0352	−0.0529
P3	0.2386	−0.3334	−0.1446	−0.0036	0.0361
P4	−0.0743	−0.261	0.4659	−0.2523	−0.0695
P5	−0.1374	−0.04	0.0666	0.6294	0.2441
P6	0.1042	0.3803	−0.2638	−0.1292	0.1344
S1	0.241	−0.0831	0.1784	−0.1148	0.4736
S2	0.295	−0.0139	0.0601	−0.1406	−0.2573
S3	0.2938	−0.0146	−0.1374	0.0916	0.0235
R1	−0.0064	0.0463	0.6222	−0.0551	−0.2513
R2	0.2825	0.1234	0.2098	0.1868	0.1927
R3	0.3037	0.0295	0.0725	0.0087	0.1144
R4	0.2807	−0.1064	−0.154	0.1125	−0.2228
R5	0.2479	−0.1222	−0.199	−0.1415	−0.1449
R6	0.1545	−0.2005	−0.1552	0.3422	−0.2919
R7	0.2131	−0.1046	0.0785	−0.1431	0.5218
R8	0.2941	−0.167	0.0367	−0.0776	0.037
R9	0.1094	0.446	0.0336	−0.0264	0.0162
R10	0.2723	−0.2201	0.0457	−0.0258	−0.1554
R11	0.1965	0.0996	0.2781	0.5122	−0.0474

After determining the five major principal component indicators, the weight of the five major components was obtained by using the entropy method of MATLAB. To facilitate readers' understanding, the five first-level indicators were named CSP, CSR, CSC, CST, and CSB, and the results are shown in Table 4.

**Table 4:** Weight calculation results of the entropy method.

**Item**	**Information entropy value e**	**Information effect value d**	**Weight coefficient w**
CSP	0.8732	0.1256	37.29%
CSR	0.9442	0.0558	16.41%
CSC	0.9469	0.0531	15.61%
CST	0.9403	0.0597	17.54%
CSB	0.9553	0.0447	13.14%

From this result, it can be seen that physical units of measurement can also be measured in entropy values. At the same time, in the case of a larger entropy value, the more chaotic the data, and the less valid information it carries, the lower the corresponding utility value, so the corresponding weight is also smaller. In addition, the research principle of the entropy value method is mainly to use the information value brought by the entropy value to determine the weight of each indicator, further analyze the indicators through the basic principles, and then bring the weights of each indicator into the description, and finally summarize the analysis results to reach a conclusion.

Through five first-level indicators: competitive sports pressure (CSP), competitive sports results (CSR), competitive sports cost (CSC), competitive sports talents (CST), competitive sports benefits (CSB) were integrated, and finally, a mathematical model for evaluating the comprehensive evaluation of the sustainable development of regional competitive sports in China was as follows:


(1) $$\begin{array}{l} CSS=0.3729CSP+0.1641CSR+0.1561CSC+0.1754CST\\ \;+0.1314CSB. \end{array}$$

Among them, CSS is a comprehensive evaluation index for the sustainable development of regional competitive sports in China. CSP, CSR, CSC, CST, and CSB are five indicators.

The standardized data of each province are brought into the comprehensive evaluation model of regional competitive sports in China. The comprehensive score of each province is obtained and the sustainable development level of competitive sports in each province is sorted according to the score to obtain the results as shown in Table 5.

**Table 5:** Comprehensive evaluation scores and ranking tables of provinces by region.

**Region**	**Province**	**Overall evaluation** **score**	**Position**
Eastern region	Guangdong	95.468	1
	Shanghai	85.158	2
	Jiangsu	82.272	3
	Beijing	75.904	4
	Shandong	72.184	5
	Zhejiang	70.623	6
	Tianjin	60.706	7
	Fujian	57.59	8
	Hebei	54.792	9
	Hainan	43.159	10
Central region	Hubei	61.059	1
	Hunan	55.268	2
	Henan	54.268	3
	Shanxi	51.689	4
	Anhui	50.631	5
	Jiangxi	47.763	6
Western region	Sichuan	61.321	1
	Shaanxi	54.213	2
	Inner Mongolia	53.323	3
	Chongqing	49.587	4
	Guangxi	47.796	5
	Yunnan	47.664	6
	Gansu	46.794	7
	Xinjiang	46.083	8
	Ningxia	42.474	9
	Guizhou	41.905	10
	Qinghai	41.01	11
	Tibet	38.648	12
Northeast region	Liaoning	63.229	1
	Heilongjiang	56.737	2
	Jilin	52.099	3

From Table 5, it can be seen that among the provinces in the eastern region of China, Guangdong has the highest comprehensive score for competitive sports, with a score of 95.468; among the provinces in the western region, the highest comprehensive score for competitive sports is Sichuan, with a score of 61.321 Among the provinces in the central region, the highest comprehensive score for competitive sports is Hubei, with a score of 61.059; among the provinces in the northeast region, the highest comprehensive score for competitive sports is Liaoning, with a score of 63.299. In addition, by comparing the scores of various regional provinces, it can be found that the provinces in the eastern region have a large advantage in the overall trend of scoring, and the other three regions and provinces are not much different. Further investigation into the reasons behind this is mainly due to the differences in the level of economic development, environmental factors, and geographical location of each region. Specifically, the economic development level in the eastern region is higher than that of the central and western regions, and most of the sports innovation talents are concentrated in the central and eastern regions; while the western regions lack talent resources and sports resources are scarce. At the same time, there are more sports in the central and eastern regions, resulting in the efficient development of the competitive sports system; while the western regions are remote due to their geographical location. There are fewer sports that can be developed, resulting in a lower level of development of competitive sports. In addition, the state's financial subsidy support for the development of sports in the eastern region is relatively large, which in turn, has a higher degree of promotion effect on the sustainable development of the competitive sports system; and the financial support for the other three regions is comparative It is not as strong as the support in the eastern region, which in turn, plays a certain role in inhibiting the sustainable development of the competitive sports system.

## Empirical Analysis of Coordinated Development Between China's Competitive Sports System and Economic Society Based on Coupling Coordination Method

Competitive sports are an important force to support economic and social development, and economic development is a booster to promote competitive sports to a higher level, and the two are closely linked. Based on the above-mentioned evaluation model, it is first necessary to standardize the data of each province, and then use the data of the secondary indicators given by each province. The principal component analysis method calculates the weights of the five first-level indicators, and then calculates the coupling coordination degree between the three subsystems of China's competitive sports and the coordinated development of the economy and society according to the pressure (P), state (S), and response (R) data of each province, and finally solves the coupling coordination degree of each province, and the specific calculation formula is as follows:


(2) $$\begin{array}{r} C={\left\{F\left(P\right)\times G\left(S\right)\times Q\left(R\right)/{\left[\frac{F\left(P\right)+G\left(S\right)+Q\left(R\right)}{3}\right]}^{3}\right\}}^{3}. \end{array}$$

In Equation (2), C is the coupling degree between the competitive sports system and the level of coordinated economic and social development. The value range is (0, 1), the size of the C value is determined by the evaluation value of the competitive sports system and the level of coordinated economic and social development, and the C value becomes larger with the enhancement of the interaction relationship between the two coordinated development levels. Where *F*(*P*), *G*(*S*), and *Q*(*R*), respectively, represent the comprehensive evaluation values of pressure, state, and response, which are calculated by means of principal component analysis in Section Introduction comprehensive evaluation values of pressure, state, and response are represented.

Drawing on the research results of existing scholars and combined with the actual situation studied in this study, the coordinated development of China's competitive sports system and economic and social development is divided into the following four types according to the value of coupling degree, as shown in Table 6.

**Table 6:** Classification of competitive sports systems and economic and social coupling types.

**The degree of coupling value**	**Coupling type**	**Features**
*C*∈(0, 0.3)	Verge of coupling out of balance	The degree of interconnectedness between the competitive sports system and the economy and society is low
*C*∈(0.3, 0.5)	Reluctantly coupled coordination	The intrinsic relevance of the three subsystems is gradually increasing
*C*∈(0.5, 0.8)	Primary coupling coordination	There is a benign coupling between the competitive sports system and the economy and society
*C*∈(0.8, 1.0)	Good coupling coordination	High level of coordination and coupling period, high degree of interconnectivity

According to the above formula, the data are imported into the SPSS software, and the coupling degree of the competitive sports system and the economic and social development of 31 provinces in China is calculated as shown in Table 7.

**Table 7:** Coupling degree between competitive sports system and economic and social development.

**Province**	**The degree of coupling value**	**Coupling type**
Beijing	0.983	I
Jiangsu	0.942	I
Guangdong	0.901	I
Shanghai	0.858	I
Liaoning	0.798	II
Zhejiang	0.795	II
Fujian	0.778	II
Shandong	0.732	II
Hubei	0.729	II
Sichuan	0.621	II
Shaanxi	0.608	II
Tianjin	0.598	II
Hebei	0.485	III
Hunan	0.463	III
Inner Mongolia	0.397	III
Jilin	0.368	III
Heilongjiang	0.342	III
Shanxi	0.292	IV
Anhui	0.283	IV
Jiangxi	0.267	IV
Henan	0.253	IV
Guangxi	0.251	IV
Chongqing	0.244	IV
Guizhou	0.231	IV
Yunnan	0.205	IV
Tibet	0.195	IV
Gansu	0.182	IV
Qinghai	0.180	IV
Ningxia	0.164	IV
Xinjiang	0.159	IV
Hainan	0.132	IV

*I: Verge of coupling out of balance; II: Reluctantly coupled coordination; III: Primary coupling coordination; IV: Good coupling coordination*.

According to Table 7, the 31 provinces in the Chinese region are divided into four types according to the intensity of interaction between the competitive sports system and the level of economic development, and only Beijing, Jiangsu, Guangdong, and Shanghai 4. The level of coupling and coordination between the competitive sports system and economic and social development in various provinces has reached a good level; Liaoning, Zhejiang, Fujian, Shandong, and Hubei have reached a good level. The coupling type of Sichuan, Shaanxi, and Tianjin is primary, indicating that the province has begun to show a trend of mutual checks and balances, and the competitive sports system and the economy and society have shown benign coupling characteristics Hebei, Hunan, Inner Mongolia, Jilin, and Heilongjiang provinces. The interaction between the competitive sports system and the economy and society has been strengthened, and the internal correlation degree of the three subsystems has gradually increased, which is manifested as a reluctant coupling and coordination type; the coupling and coordination degree between the competitive sports system and the economy and society in the remaining 14 provinces is on the verge of being out of balance. Therefore, from the above analysis, it can be found that most provinces in the eastern region have developed well in coordination with the economic level, followed by the central region, and the northeast and the western region are relatively backward, that is, the coupling coordination level shows a decreasing trend of the eastern → the central → the northeast → the western part.

Comprehensive analysis of the reasons for the occurrence of the above phenomena is mainly in the following aspects: for the eastern region, first, the eastern region is mostly located in the coastal area, with a high level of economic development, strong innovation ability, rich sports resources, and the development of competitive sports. The development provides a solid foundation. Second, the state has invested more in the economy and material resources of the eastern region, attracting a large number of talents, especially sports talents, making the coordinated development of competitive sports and economic levels more cutting-edge. Third, the residents of the eastern region are more open-minded, can well accept the rapid development of competitive sports, and at the same time realize the inextricable relationship between competitive sports and the level of economic development, which can effectively promote the improvement of their living standards and then coordinate them the level of development works better. For the central region, the current economic development level is in a period of transition, not as active as the eastern region, and at the same time, affected by the geographical location. Many high-end sports talents are reluctant to come to the central region, resulting in the coupling and coordination between the competitive sports system and the economic level being not as good as in the eastern region. For the northeast region, the development of competitive sports talents is not comprehensive enough. Due to the geographical location of the northeast region, most of the athletes trained are engaged in ice and snow sports, such as the 2022 Beijing Winter Olympics. Many Olympic athletes in China are from the northeast region, such as Wu Dajing, Jin Boyang, and so on. Although the level of sports development in the northeast of China is significantly better than that of other regions for Winter Olympics, the athletes in the northeast region are slightly inferior to the more well-known Olympic Games than the Winter Olympic Games. Second, the level of sports development does not match the level of economic development. The overall level of sports development in the eastern region is high, but the overall level of economic development is low, which leads to a low degree of coupling and coordination between the two. First, the geographical location of the western region is relatively remote, the level of economic development is backward, and the lack of sports resources has led to the development level of competitive sports in the western region lagging behind that of the central and eastern regions. Second, the state has invested less in the development of sports in the western region. Its innovation ability is weak and the development of the local competitive sports system has not been able to keep up with the pace of the development of the international competitive sports system in time, making it difficult to attract sports innovation talents. Third, the thinking of residents in the western region is relatively conservative, and they have not fully accepted the new idea that competitive sports have gradually become the sports of the whole people, resulting in a deviation in the overall residents' cognition of competitive sports, which in turn, has limited the level of coupling and coordinated development between the competitive sports system and the economic level.

## Obstacles to Sustainable Development of China's Competitive Sports

### Research Ideas

After research and analysis of the development status of competitive sports in various provinces in China and its coupling and coordination with economic and social development to promote the efficient and sustainable development of competitive sports, the main obstacles to the sustainable development of competitive sports will be studied below. First of all, the barrier degree formula is used to calculate the obstacle degree of each factor in each province, and the second is that all provinces are divided into four regions: the eastern, central, western, and northeastern regions. At the same time, to link the provinces in the four regions, this study uses the gray association analysis method. It uses the degree of an obstacle as an indicator for analysis, and finally ranks the degree of an obstacle of each factor to analyze the main obstacle factors affecting the sustainable development of competitive sports in various regions.

### Model Creation

#### Establishment of Diagnostic Model for Disorder Factors

The barrier factor diagnosis model analyzes the data of various indicators of competitive sports in various regions and calculates the degree of obstacle of each factor from the two levels of indicator categories and indicators, to determine the current factors hindering sustainable development of competitive sports in various regions. The blocking factor diagnostic model is calculated as follows:


(3) $$\begin{array}{r} I_{ij}=1-Z_{ij}. \end{array}$$

(4) $$\begin{array}{r} A_{ij}=\frac{I_{ij}G_{j}}{\sum_{j=1}^{20}I_{ij}G_{j}}. \end{array}$$

In the above formula, *I*_*ij*_ is the deviation degree of the JTH index of the ith sample; *Z*_*ij*_ is the fitting degree of a single index; *G*_*j*_ is the weight of the JTH index in the evaluation system; and *A*_*ij*_ is the hindrance degree of the JTH index of the ith sample.

#### Establishment of Gray Association Analysis Model

The gray correlation analysis method is used in supplier selection decision-making for a large number of uncertainties and their interrelationships, and organically combines quantitative and qualitative methods to make the original complex decision-making problems clearer and simpler, and the calculation is convenient and can be used to a certain extent. The subjective arbitrariness of decision-makers is excluded, and the conclusions drawn are more objective and have a certain reference value. The specific steps for the establishment of the gray association analysis model are as follows:

(1) Determine the comparator (evaluation object) and the reference number column (evaluation criteria). There are m evaluation objects, n evaluation indicators, reference numbers listed, *x*_0_{*x*_0_(*k*)|*k* = 1, 2, ⋯, *n*} and comparative numbers listed as columns *x*_*i*_{*x*_*i*_(*k*)|*k* = 1, 2, ⋯, *n*}, *i* = 1, 2, ⋯*m*.
(2) Determine the weight corresponding to the value of each indicator. The weight corresponding to each index can be determined by using the analytic hierarchy method ω = [ω_1_, ⋯, ω_*n*_], of which the weight corresponding to the ω_*k*_(*k* = 1, 2, ⋯, *n*) k-th evaluation index is used.
(3) Calculate the gray correlation coefficient:

(5) $$\begin{array}{r} \xi_{i}\left(k\right)=\frac{\underset{s}{\min}\underset{t}{\min}|x_{0}\left(t\right)-x_{s}\left(t\right)|+\rho \underset{s}{\max}\underset{t}{\max}|x_{0}\left(t\right)-x_{s}\left(t\right)|}{\left|x_{0}\left(k\right)-x_{i}\left(k\right)|+\rho \underset{s}{\max}\underset{t}{\max}|x_{0}\left(t\right)-x_{s}\left(t\right)\right|}. \end{array}$$

The correlation coefficient listed on the *x*_*i*_*x*_0_ k-th indicator for the comparison sequence pairs the reference number, where is the resolution coefficient. Among them, the two-stage minimum difference and the two-stage maximum difference are said to be, respectively, $\rho \;\in \;[0,\;1]\;|x_{0}(t)\;-\;x_{s}\;\left(t\right)\;|\max_{s}\;\left|x_{0}\;\left(t\right)\;-x_{s}\;\left(t\right)\;\;\right|$.

Generally speaking, the higher the resolution coefficient is, the higher the resolution is. The smaller the ρ, the smaller the resolution.

(4) Calculate the gray weighted correlation degree. Gray weighted correlation degrees are calculated as:

(6) $$\begin{array}{r} r_{i}=\sum_{k=1}^{m}\omega_{i}\xi_{i}\left(k\right). \end{array}$$

Where *r*_*i*_ is the gray weighted correlation degree of the i-th evaluation object to the ideal object.

### Solve the Barrier Factors for Each Region According to the Gray Association Method

According to the 20 indicators of “pressure-state-response” included in the PSR model above, in this study, 20 indicators are used as factors hindering competitive sports in various regions, and the comprehensive score of each index in each region is solved according to the obstacle factor diagnosis model and the gray correlation method, and the higher the score, the greater the obstruction of the index to the region. For example, in the east of China area, this study first of all, the region will be 20 indicators data in turn into obstacle factor model, the solution of each factor blocking degree, at the same time to will be linked to the provinces in the four regions, this article USES the gray correlation analysis method, using degree as index were analyzed, and all indexes have to sort of get Table 8.

**Table 8:** Obstacle scores and rankings by region.

**Region**	**Index**	**Score**	**Ranking**
East division earth district	S1	0.8386	1
	R6	0.8026	2
	R11	0.7941	3
	P6	0.7829	4
	R7	0.727	5
	P5	0.6738	6
	R8	0.6551	7
	P3	0.6434	8
	R2	0.6432	9
	P4	0.6332	10
	R3	0.6187	11
	R4	0.617	12
	R1	0.6089	13
	R10	0.6059	14
	R5	0.5564	15
	S2	0.5558	16
	S3	0.5489	17
	P1	0.5232	18
	P2	0.5188	19
	R9	0.5031	20
Middle division earth district	R11	0.8431	1
	S3	0.7337	2
	R3	0.7165	3
	P1	0.714	4
	P6	0.7117	5
	P4	0.6911	6
	R1	0.6899	7
	R9	0.684	8
	S2	0.6516	9
	R2	0.6393	10
	R4	0.6368	11
	R10	0.628	12
	P3	0.6134	13
	P5	0.5917	14
	R7	0.5752	15
	R8	0.5669	16
	S1	0.5667	17
	P2	0.5562	18
	R6	0.5466	19
	R5	0.5042	20
West division earth district	R8	0.8254	1
	S1	0.7879	2
	R11	0.7682	3
	R5	0.7146	4
	S3	0.6849	5
	R6	0.6758	6
	S2	0.6734	7
	P3	0.6466	8
	R4	0.613	9
	P4	0.6127	10
	R10	0.6098	11
	R1	0.6096	12
	R2	0.6055	13
	R3	0.5985	14
	P6	0.5903	15
	R9	0.5688	16
	P1	0.5639	17
	R7	0.5571	18
	P5	0.5527	19
	P2	0.4882	20
East north earth district	P4	0.7778	1
	R5	0.7684	2
	R6	0.7653	3
	R2	0.7539	4
	S1	0.7511	5
	R1	0.7225	6
	R7	0.7017	7
	P6	0.6948	8
	P2	0.6572	9
	R4	0.613	10
	R3	0.6076	11
	S2	0.5897	12
	P3	0.5895	13
	R9	0.5864	14
	P5	0.5851	15
	R10	0.5809	16
	R11	0.5789	17
	R8	0.5735	18
	P1	0.5636	19
	S3	0.5616	20

**Figure 5:**
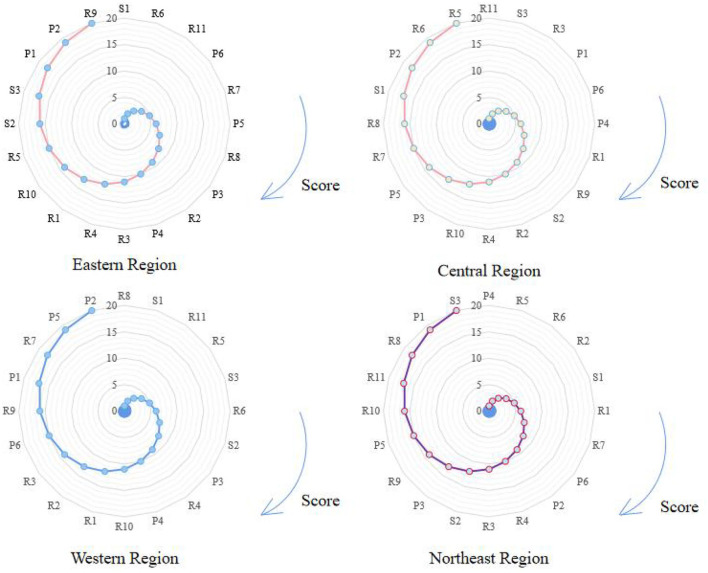
Gray correlation degree of barriers for various indicators in various regions.

From the above solution results, the main obstacle factor can be selected according to the score of each obstacle factor, and the top five obstacle factor scores in each region are taken as the main obstacle factors in this area. Therefore, the main obstacle factors in the eastern region are the number of athletes who have won the World/Olympic championship (S1), the number of referees (R6), the number of scientific research funds (R11), the proportion of illiterate people over 15 years old (P6), and the number of youth sports clubs (R7). The main obstacle factors in the central region are access to scientific research grants (R11), the number of athletes on excellent sports teams (S3), cultural and sports media expenditure (R3), GDP per capita (P1), and the proportion of illiterate people over 15 years old (P6). The main obstacle factors in the western region are the number of schools in traditional sports (R8), the number of athletes who have won World/Olympic championships (S1), the number of scientific research funds (R11), the number of sports reserve talents (R5), and the number of athletes on outstanding sports teams (S3). The main obstacle factors in the northeast region are the natural population growth rate (P4), the number of sports reserve talents (R5), the number of referees (R6), the public budget expenditure of the sports system (R2), and the number of athletes who have won world/Olympic champions (S1). Figure 5 shows the gray correlation degree of obstacles for each indicator in each region.

According to the gray correlation degree of the index obstacle in each region, it can be found that the top five obstacle factors in different regions are different, and there are also differences in the obstacle scores. For example, in the eastern region with a good level of competitive sports development, when some hard resources are reduced, such as the number of world/Olympic champion athletes, the number of referees, and the number of scientific research funds, the development of scientific research funds and other resources are reduced. It will hinder the development of eastern competitive sports. The first is the human resource factor. Competitive sports itself is a sport that relies on human development and the number of champion athletes and the number of referees will directly affect the development of competitive sports. Second, the factor of scientific research funding. The amount of government investment in sports in the eastern region will also directly affect its development, and when the amount of funding declines, it will hinder the development of competitive sports in the eastern region. The same is true of the other three regional analysis principles, although the difference is that the degree of resistance of each factor is different, and there is regional heterogeneity.

## Prediction of the Future Development of Competitive Sports Based on the DEA Model

After understanding the current situation, coordinated development and hindering factors of competitive sports development in various provinces and regions, the following article will predict the sustainable development of competitive sports in China's provinces in the next 10–20 years. In this study, the model in the data envelopment analysis will be used, and the pressure and response of the *C*^2^*R* PSR model in the previous article will be used as the input index and the state as the output index, to evaluate the future level of sports development and obtain the level of development of scale remuneration.

Suppose there are n DMU, and each DMU has m kinds of inputs and S kinds of outputs. Let *x*(*i* = 1, ⋯, *m*; *j* = 1, ⋯, *n*) represent the input quantity of the ith kind of the JTH DMU, *y*_*rj*_ = (*r* = 1, ⋯, *s*; *j* = 1, ⋯*n*) represent the output quantity of the RTH kind of the JTH DMU, *v*_*i*_(*i* = 1, ⋯, *n*) represent the weight of the input of the ith kind, and *j*_*r*_(*r* = 1, ⋯*s*) represent the weight of the output of the RTH kind.

Vector _*X*_*j*_, *Y j*_(*j* = 1, …, *n*) represents the input and output vectors of decision unit J, respectively, and v and u represent the input and output weight vectors, respectively, $X_{j}\;=\;(x_{1j},x_{2j},\cdots ,x_{mj})'Y_{j}\;=\;(y_{1j},y_{2j},\cdots ,y_{ij})'u_{j}\;=\;(u_{1},u_{2},\cdots ,u_{m})'v_{j}\;=\;\;(v_{1},v_{2},\cdots ,v_{s})'$.

The efficiency evaluation index of the defined decision unit j is:


(7) $$\begin{array}{r} h_{j}=\left(u^{T}Y_{j}\right)/\left(v^{T}X_{j}\right),j=1,2,\cdots \;,n. \end{array}$$

The mathematical model for evaluating the efficiency of the decision unit is:


(8) $$\begin{array}{r} \max\frac{u^{T}Y_{j0}}{v^{T}X_{j0}}, \end{array}$$

(9) $$s.t.\{\begin{array}{c} \frac{u^{T}Y_{j0}}{v^{T}X_{j0}}\leq 1,j=1,2,\cdots n,\\ u\geq 0,v\geq 0,u\neq 0,v\neq 0. \end{array}$$

With the Chaarnes-Cooper transform, the above model can be transformed into an equivalent linear programming problem $\omega =tv,u=tu,t=\frac{1}{v^{T}X_{j0}}$ as follows:


(10) $$\begin{array}{l} \;\max V_{j0}=u^{T}Y_{j0}.\\ s.t.\{\begin{array}{c} \omega^{T}X_{j}-u^{T}Y_{j}\geq 0,j=1,2,\cdots ,n,\\ \omega^{T}X_{j0}=1,\\ \omega \geq 0,u\geq 0. \end{array} \end{array}$$

It can be shown that model (9) is equivalent to model (10). The dual linear programming model of the linear programming problem has clear economic significance. The following is the dual form of the model (11):


(11) $$\begin{array}{l} \;min\theta .\\ s.t.\{\begin{array}{c} \sum_{j=1}^{n}\lambda_{j}X_{j}\leq \theta X_{j0},\\ \sum_{j=1}^{n}\lambda_{j}Y_{j}\geq Y_{j0},\\ \lambda_{J}\geq 0,j=1,2,\cdots ,n. \end{array} \end{array}$$

According to the algorithm, the technical efficiency value and the pure technical efficiency value are calculated with known data, and the sum of the two is calculated to predict the scale income of each province, and when the scale income of the province increases, it indicates that the province has a better development of competitive sports in the next 10–20 years. The results of the specific scale income of each province are shown in Table 9.

**Table 9:** Scale gains by province.

**Province**	TEV^*^	PEV^**^	**Scale gains**
Beijing	1	1	Unchanged
Tianjin	1	1	Unchanged
Hebei	1	1	Unchanged
Shanghai	1	1	Unchanged
Jiangsu	1	1	Unchanged
Chekiang	1	1	Unchanged
Fujian	1	1	Unchanged
Shandong	1	1	Unchanged
Guangdong	1	1	Unchanged
Hainan	0.672	1	Increasing
Shanxi	0.885	1	Increasing
Anhui	0.781	1	Increasing
Jiangxi	0.729	1	Increasing
Henan	1	1	Unchanged
Hubei	1	1	Unchanged
Hunan	1	1	Unchanged
Inner Mongolia	0.977	1	Increasing
Guangxi	1	1	Unchanged
Chongqing	1	1	Unchanged
Sichuan	1	1	Unchanged
Guizhou	1	1	Unchanged
Yunnan	1	1	Unchanged
Tibet	1	1	Unchanged
Shaanxi	1	1	Unchanged
Gansu	0.739	0.755	Increasing
Qinghai	1	1	Unchanged
Ningxia	0.844	1	Increasing
Xinjiang	1	1	Unchanged
Liaoning	1	1	Unchanged
Jilin	1	1	Unchanged
Heilongjiang	1	1	Unchanged

* *TEV is an abbreviation for technical efficiency values*,

** *PEV is an abbreviation for Pure technical efficiency values*.

From the above prediction results, it can be seen that Hainan, Shanxi, Anhui, Jiangxi, Inner Mongolia, Gansu, and Ningxia will be 20 in the next 10 years. The increase in the annual scale of income indicates that the development of competitive sports in these provinces is better in the future and has a large room for growth. The unchanged scale income of other provinces indicates that the development of competitive sports in these provinces in the next 10–20 years is relatively unchanged, mainly due to the fact that in these provinces and Hainan and Shanxi, compared with Anhui, Jiangxi, Inner Mongolia, Gansu, and Ningxia, the increase in scale income is small.

Based on the prediction results, we delve into the reasons behind it. In recent years, a number of policies promulgated by China have repeatedly emphasized the coordinated development of various regions and balanced the incoordination between regions, and the development trend of competitive sports in the next 10–20 years is also moving toward this aspect, which is in line with the current and future economic and social development of the era background. At present, the level of competitive sports development in the central and western regions is not as good as that in the eastern region mainly in terms of human and material resources, there is a certain lag. When the resources have been balanced, the government departments to improve the resource supplement to the central and western regions, its future development utility will be greatly improved compared with the present, there has been an increase in scale efficiency; and the current level of competitive sports development in the eastern region is good, and the resources in all aspects are close to being satisfied. The marginal benefits of continuous transportation of resources are not significant and are manifested in the same fluctuations as the current level of competitive sports development, so the scale benefits brought by it are close to the same. In summary, in the coming time, there is a large room for improvement in the development level of competitive sports in the central and western regions, and various government departments and sports departments should pay attention to and increase the allocation of resources to the central and western regions to promote the coordinated development of competitive sports between regions and ensure the sustainable development of the entire Chinese competitive sports system.

## Conclusion

In today's era of rapid development, competitive sports have become an important indicator to measure the level of China's comprehensive strength. The sustainable development of competitive sports has important practical value and theoretical significance for promoting China's economic development, stimulating patriotic feelings, and carrying forward the spirit of Chinese civilization. In this study, based on constructing the indicators of the comprehensive development level of competitive sports, the PSR model, the coupled coordination method, the obstacle factor method, and the DEA model are used to align. The development status of national competitive sports was evaluated at a comprehensive level and the coordinated development of competitive sports and economic and social development was studied. The obstacles to the sustainable development of China's competitive sports system were studied, and the future 10-2 was studied. The 0-year development situation is predicted. The main conclusions obtained are as follows:

(1) From the perspective of the overall macro pattern of China, the overall development level of China's competitive sports development level is good, but there are certain differences between various regions. Specifically, the level of competitive sports development in various provinces in the eastern region is higher than that in the other three regions, and in the eastern region, Guangdong Province has the best level of competitive sports development. There is little difference in the development level of competitive sports in the western region, the central region, and the northeast region, and the provinces corresponding to the highest level of competitive sports development in each region are Sichuan Province, Hubei Province, and Liaoning Province.
(2) From the perspective of the degree of coordination between China's competitive sports and economic development level, only the level of coupling and coordination between the competitive sports system and economic and social development in Beijing, Jiangsu, Guangdong, and Shanghai has reached a good level; the coupling type of Liaoning, Zhejiang, Fujian, Shandong, Hubei, Sichuan, Shaanxi, and Tianjin is primary, indicating that the provinces have begun to show a trend of mutual checks and balances; Hebei, Hunan, Inner Mongolia, The interaction between the competitive sports system and the economy and society in the five provinces of Jilin and Heilongjiang has been strengthened; the coupling and coordination degree between the competitive sports system and the economy and society in the remaining 14 provinces is on the verge of imbalance.
(3) From the perspective of the obstacle factors hindering the sustainable development of competitive sports in China, there are differences in the hindering factors in different regions. The main obstacle factors in the eastern region are the number of champion athletes, the number of referees developed, and the scientific research funds; the main obstacle factors in the central region are scientific research funds, the number of athletes of outstanding sports teams, and the expenditure on culture, sports, and media; the main obstacle factors in the western region are the number of schools in traditional sports projects, the number of champion athletes, and the number of scientific research funds; the main obstacle factors in the northeast region are the natural population growth rate, the number of sports reserve talents, and the number of referee development.
(4) Judging from the prediction results of China's competitive sports in the next 10–20 years, Hainan, Shanxi, Anhui, Jiangxi, Inner Mongolia, Gansu, and Ningxia—these seven provinces in the competitive sports may have a large level of improvement and the room for improvement is larger. Other provinces in recent years the development of competitive sports may be close to saturation, so it is difficult to have a more obvious improvement in the future, that is, compared with the above provinces, the development space is small, the scale benefits are low.

To ensure high quality and sustainable development of China's competitive sports, this study puts forward the following three policy suggestions based on the above analysis and conclusions. First, we should give full play to the effects of joint and synergistic development among different regions. To form a unified internal mechanism in education, science and technology and personnel training, and promote the coordinated development of competitive sports in multiple regions. Provincial governments should recognize the shortcomings of their own development while affirming the development of competitive sports in their own regions, and strengthen the cooperation with other regions to develop competitive sports in each region into the spirit of China's great power (He et al., 2022; Zhu et al., 2022). The results of empirical analysis show that the development level of competitive sports in the eastern region is better than that in the central and western regions. Therefore, the central and western regions should learn from the eastern region to promote the coordinated development of various regions. Second, draw on the advanced experience in the development of competitive sports at home and abroad. Based on the results of the empirical analysis, this article 10–20 years for China's future development of competitive sports horizontal space is larger, but want to make the high quality and sustainable development of competitive sports in China, our country needs to continue to optimize sports team internal structure, improve the level of the regional development of competitive sports, draw lessons from various countries to implement the effective policies and programs. For example, the Incentive policies issued by the United States for competitive sports athletes include education, life, and hobby cultivation from childhood. China can carry out reasonable reform and innovation according to the national conditions of China, constantly improve the development level of competitive sports, and achieve high quality and sustainable development of regional competitive sports. Third, we will scientifically build a comprehensive development system for competitive sports. In the research results of this study, there is heterogeneity in the development of competitive sports in different provinces. One of the main reasons is the unreasonable allocation of resources among regions. Therefore, to maximize the effectiveness of regional sports, each region should jointly build a set of comprehensive evaluation sports to measure the development of its various indicators and form a fair evaluation mechanism for the development of competitive sports in each region according to the evaluation system. This measure can effectively promote the allocation of resources in each region to optimize the allocation of resources and realize the efficient development of competitive sports.

## Data Availability Statement

The original contributions presented in the study are included in the article/supplementary material, further inquiries can be directed to the corresponding author.

## Author Contributions

All authors listed have made a substantial, direct, and intellectual contribution to the work and approved it for publication.

## Funding

This study was funded by the National Social Science Fund Project of China (21CTJ024), Teaching, Humanities and Social Sciences Research Major Project of Education Department of Anhui Province (SK2019A1161), and Research Project of Anhui University of Finance and Economics (acxkjs2021005 and acyljc2021002).

## Conflict of Interest

The authors declare that the research was conducted in the absence of any commercial or financial relationships that could be construed as a potential conflict of interest.

## Publisher's Note

All claims expressed in this article are solely those of the authors and do not necessarily represent those of their affiliated organizations, or those of the publisher, the editors and the reviewers. Any product that may be evaluated in this article, or claim that may be made by its manufacturer, is not guaranteed or endorsed by the publisher.

## References

[B1] Cai X.-W. Bao Y.-Q. Hu M.-F. (2021). Simulation and prediction of fungal community evolution based on RBF neural network. Comput. Math. Methods Med. 2021, 7918192. 10.1155/2021/7918192 34659448 PMC8519688

[B2] Chun L.-H. Chun L.-W. (2011). On the sustainable development of competitive sports in ‘post olympic' period of olympic hosting countries-based on system regulation theory. Adv. Mater. Res. 50, 225–226. 10.4028/www.scientifific.net/AMR.225-226.132

[B3] Cui X.-Y. Yin H.-G. (2022). The Chinese dream and the Olympic wind: a review of the academic workshop on ice and snow is also a golden mountain and a silver mountain: the late effect of the Beijing winter Olympics. Sports Sci. 43, 8–16. 10.13598/j.issn1004-4590.2022.02.001

[B4] Dong J.-H. (2015). The enlightenment of the legalization of the training of reserve talents in foreign competitive sports to China. J. Shenyang Instit. Phys. Educ. 22, 54–58. 10.3969/j.issn.10040560.2015.05.011

[B5] Du C.-X. Wu J.-L. Gao Y. (2020). A comparative study of sports governance systems in the United States, Britain, Australia and China. J. Phys. Educ. Adult Educ. 25, 71–76. 10.16419/j.cnki.42-1684/g8.2020.03.015

[B6] He Q. Tong H. Liu J. B. (2022). How does inequality affect the residents' subjective well-being: Inequality of opportunity and inequality of effort. Front. Psychol. 13 (2022). 10.3389/fpsyg.2022.843854 35465572 PMC9019070

[B7] He Q. Z. Xia P. T. Li B. Liu J-B. (2021). Evaluating investors? recognition abilities for risk and profit in online loan markets using nonlinear models and financial big data. J. Funct. Spaces. 2021, 5178970. 10.1155/2021/5178970

[B8] Hou X.-C. Xiao K.-P. (2021). Integration of sports and education: collaborative governance of reserve talent training in youth competitive sports. J. Shenyang Univ. Phys. Educ. 40, 90–97. 10.12163/j.ssu.20210173

[B9] Huang T.-T. (2021). Study on the sustainable development of competitive sports in China based on PSR Model. Sci. J. Econ. Manag. Res. 3, 31–37.

[B10] Lei X.-Y. Li Y.-J. Xie Q.-W. Liang L. (2014). Measuring olympics achievements based on a parallel DEA approach. Ann. Oper. Res. 226, 379–396. 10.1007/s10479-014-1708-1

[B11] Li S.-L. Li L.-T. (2014). Research on evaluation model of competitive sports efficiency in provinces and regions of China. J. Chengdu Sport Univ. 2, 26–32. 10.3969/j.issn.1001-9154.2014.02.005

[B12] Li Y. (2008). Models for measuring and benchmarking Olympics achievements. Omega. 36, 933–940. 10.1016/j.omega.2007.05.003

[B13] Liu Y.-Y. (2011). Research on the Training Model of Competitive Sports Talents in China in the Period of Social Transformation. Hunan Normal University, Changsha, China.

[B14] Lu Y. Shi S.-S. (2021). Thinking turn and approach selection of physical-educational integration under public service perspective. J. Tianjin Univ. Phys. Educ. 36, 652–657+681. 10.13297/j.cnki.issn1005-0000.2021.06.005

[B15] Man J.-H. (2018). Dynamic simulation research of sports enterprise collaborative path optimization system based on DEA. J. Xi'an Sports Univ. 2, 10–22. 10.16063/j.cnki.issn1001-747x.2018.01.002

[B16] Peng G.-Q. Yang G.-Q. (2021). Development strategy and innovation path of China's competitive sports during the 14th five-year plan period. J. Capit. Univ. Phys. Educ. 12, 257–267. 10.14036/j.cnki.cn11-4513.2021.03.004

[B17] Qu Y.-C. Gao L.-X. (2022). The training model of foreign competitive sports talents and its enlightenment (I)–a case study of the United States and Australia. J. Nanjing Univ. Phys. Educ. (Natl. Sci. Ed.). 24, 54–58. 10.15877/j.cnki.nsin.2017.05.012

[B18] Su Y. (2020). Research on the connotation of Jin.-Ping. Xi People-centered and Big Sports Concept. J. Nanjing Univ. Phys. Educ. 19, 30–33. 10.15877/j.cnki.nsin.2020.12.005

[B19] Sun M.-K. Sun X.-L. Gong L.-J. Sun Y.-P. (2021). The development model of ice and snow sports in Japan and its enlightenment to the leapfrog development of ice and snow sports in China. J. Wuhan Univ. Phys. Educ. 55, 28–34. 10.15930/j.cnki.wtxb.2021.12.005

[B20] Sun M.-K. Sun X.-L. Gong L.-J. Sun Y.-P. (2022). South Korea's experience and reference for hosting the winter Olympics to promote the development of ice and snow sports. J. Beijing Sport Univ. 45, 158–168. 10.19582/j.cnki.11-3785/g8.2022.01.016

[B21] Wang L.-Y. (2013). A Study on the Corruption of Competitive Sports in China in the Transition Period. Beijing Sport University.

[B22] Wang S.-B. (2019). Research on dilemma and breakthrough path of China's competitive sports development strategy. J. Beijing Sport Univ. 8, 72–81+101. 10.19582/j.cnki.11-3785/g8.2019.10.009

[B23] Xia L.-P. (2022). Sports diplomacy and the winter Olympics from the perspective of a community with a shared future for mankind. Contemp. World. 15, 15–20. 10.3969/j.issn.1006-4206.2022.02.005

[B24] Xu F. Mo L.-Y Chen H. Zhu J.-M. (2021). Genetic algorithm to optimize the design of high temperature protective clothing based on BP neural Network. Front. Phys. 2021, 600564. 10.3389/fphy.2021.600564

[B25] Yang G.-Q. (2022). The development of competitive sports in China and the relief strategy. J. Shanghai Univ. Sport. 46, 1–9. 10.16099/j.sus.2021.07.06.0002

[B26] Yang G.-Q. Peng G.-Q. (2018). Research on the strategic mission and innovation path of China's competitive sports in the new era. Sports Sci. 5, 3–14+46. 10.16469/j.css.20180900

[B27] Yang H.-D. Li C.-X. (2021). The value, dilemma and path of digital sports boosting the construction of a sports power. Sports Cult. Guide. 34, 1–6. 10.3969/j.issn.1671-1572.2021.12.002

[B28] Yang Y.-W. Huang C.-H. (2015). Systematic structure analysis of sustainable development of reserve talent training in competitive sports in China. J. Capit. Univ. Phys. Educ. 21, 151–155. 10.14036/j.cnki.cn11-4513.2015.02.012

[B29] Ye S.-Z. (2020). A study on the coupling coordination degree of regional sports industry development and healthy China construction: a case study of 11 provinces and cities in the eastern region of China. J. Wuhan Univ. Phys. Educ. 54, 31–40. 10.15930/j.cnki.wtxb.2020.05.005

[B30] You G.-P. Liu H.-R. Zhang C.-H. Wang D.-X. (2016). Research on training benefit of competitive sports in different regions of China based on DEA Model. China Sports Sci. Technol. 4, 26–33. 10.16470/j.csst.201605004

[B31] Yu J. Ye L. (2021). An empirical analysis of the sustainable economic development of competitive sports in china based on principal component and spatial coupling coordination method. J. Xinjiang Normal Univ. (Nat. Sci. Ed.). 40, 54–58. 10.14100/j.cnki.1008-9659.2021.02.014

[B32] Yu S.-M. Wang X.-Y. (2021). Theoretical connotation and practical exploration of cultivating new talents in the era to promote the construction of a sports power. J. Phys. Educ. 28, 1–7. 10.16237/j.cnki.cn44-1404/g8.2021.06.001

[B33] Zhang L.-J. Sun Y.-P. (2021). Economic logic and moral dilemma: on the ethical dilemma and possible way out of the commercialization of competitive sports. Sports Sci. 41, 88–97. 10.16469/j.css.202107010

[B34] Zhang S.-M. Zhan W.-L. Hu H. Liu Y.-S. Zhu J.-M. (2022). Research on ethanol coupling to prepare C_4_ olefins based on BP neural network and cluster analysis. J. Chem. 2022, 10. 10.1155/2022/5324336

[B35] Zhou F.-C. (2019). Research on the Function of Competitive Sports in China. Jiangxi Normal University, Nanchang, China.

[B36] Zhu H.-H. (2018). The Impact of the National System on Competitive Sports in China. China University of Mining and Technology, Xuzhou, China.35755760

[B37] Zhu J.-M. Zhu Y.-G. Geng W.-B. Li X. L. He Q.-Z. (2022). Fuzzy decision-making analysis of quantitative stock selection in vr industry based on random forest model. J. Func. Space. 2022, 7556229. 10.1155/2022/7556229

[B38] Zou Y.-H. Tian S. (2020). An empirical study on the impact of social security on investment efficiency of competitive sports based on dynamic DEA-SBM Model. J. Wuhan Univ. Phys. Educ. 3, 50–57. 10.15930/j.cnki.wtxb.2020.10.007

